# ERRα suppression enhances the cytotoxicity of the MEK inhibitor trametinib against colon cancer cells

**DOI:** 10.1186/s13046-018-0862-8

**Published:** 2018-09-05

**Authors:** Sheng Zhou, Hongwei Xia, Huanji Xu, Qiulin Tang, Yongzhan Nie, Qi yong Gong, Feng Bi

**Affiliations:** 1 Department of Medical Oncology, Cancer Center, West China Hospital, Sichuan University, Sichuan Province, Chengdu, China; 2 Laboratory of Molecular Targeted Therapy in Oncology, State Key Laboratory of Biotherapy and Cancer Center, West China Hospital, Sichuan University, Sichuan Province, Chengdu, China; 3 Collaborative Innovation Center for Biotherapy, Sichuan Province, Chengdu, China; 4 State Key Laboratory of Cancer Biology & Xijing Hospital of Digest Diseases, Fourth Military Medical University, Xi’an, Shanxi Province China; 5 Department of Radiology, West China Hospital, Sichuan University, Sichuan Province, Chengdu, China; 6 Laboratory of Molecular Targeted Therapy in Oncology/Department of Medical Oncology, West China Hospital, Sichuan University, Sichuan Province, Chengdu, 610041 China

**Keywords:** Oestrogen-related receptor α (ERRα), Epidermal growth factor (EGF), Trametinib, Simvastatin

## Abstract

**Background:**

ERRα, a constitutive transcription factor that regulates energy metabolism, plays an important role in the progression of various tumours. However, its role in cell survival and proliferation and its implication in targeted therapy in colon cancer remains elusive.

**Methods:**

The expression of ERRα in colon cancer tissues and cell lines was detected by using western blotting and immunohistochemistry. A wound healing assay and a transwell assay were performed to examine the migration and invasion of the colon cancer cells. A cell viability assay, clonogenic assay, western blot assay and the dual-luciferase reporter assay were employed to study the interaction between trametinib (inhibitor of MEK) and EGF treatment. Flow cytometry, western blotting, quantitative reverse-transcription polymerase chain reaction and xenograft studies were used to identify whether the combination of trametinib and simvastatin had a synergistic effect.

**Results:**

ERRα positively regulated the cell proliferation, migration and invasion of colon cancer cells, and the suppression of ERRα completely reduced the EGF treatment-induced proliferation of colon cancer cells. Further investigation showed that trametinib partially restrained the up-regulation of ERRα induced by the EGF treatment, and ERRα inhibition increased the sensitivity of colon cancer cells to trametinib. At last, we combined trametinib with simvastatin, a common clinically used drug with a new reported function of transcriptional activity inhibition of ERRα, and found that this combination produced a synergistic effect in inhibiting the proliferation and survival of colon cancer cells in vitro as well as in vivo.

**Conclusions:**

The present data indicated that ERRα acted as an oncogene in colon cancer cells, and the combined targeting of ERRα and MEK might be a promising therapeutic strategy for colon cancer treatment.

**Electronic supplementary material:**

The online version of this article (10.1186/s13046-018-0862-8) contains supplementary material, which is available to authorized users.

## Background

Colon cancer is the third most common and second deadliest malignancy in the word [1]. Chemotherapy and targeted therapy remain the key strategies for the treatment of the metastatic colon cancers. However, due to the mutation and over-expression of EGFR/RAS/BRAF, the abnormal activation of EGFR/RAS pathway occurs frequently in colon cancers and is associated with a poor prognosis and drug resistance [2, 3].

EGFR plays a critical role in the process of proliferation and differentiation in colon cancer cells. Activated EGFR constitutively activates multiple downstream pathways, including the RAS/MEK/ERK (MAPK-extracellular signal regulated kinase) and AKT/PI3K/mTOR pathways [4]. Various target drugs, including cetuximab, bevacizumab and regorafinib, are widely used in colon cancer and are involved in targeting the EGFR signalling molecules. However, their effects remain limited. A number of preclinical therapeutic strategies have been developed by combining EGFR pathway inhibitors with other target drugs in BRAF/KRAS mutant colon cancers [5–7]. However, none of these have been approved for clinical use because of safety issues or a lack of objective responses. Thus, it is urgent for us to develop more robust therapeutic approaches for the treatment of colon cancers.

Trametinib, a highly specific and potent MEK1/2 inhibitor, is approved by the Food and Drug Administration (FDA) for the treatment of BRAF-mutated metastatic melanoma. The dual inhibition of BRAF and MEK was tested in patients with metastatic BRAFV600E colon cancers but showed little efficacy [8].

The orphan nuclear hormone receptor, oestrogen-related receptor A (ERRα, NR3B1), is a constitutive transcription factor that is structurally and functionally related to the classic oestrogen receptors [9]. It interacts with and is modulated by members of the SRC and PGC-1 families of co-activators [10–13]. Moreover, ERRα’s target genes include its own gene ESRRA [12], and it participates in the regulation of mitochondrial biogenesis and energy metabolism [13–16]. ERRα plays an important role in the carcinogenesis of various tumours. A high expression of ERRα is globally associated with a poor prognosis in colon, endometrium, ovary, breast and prostate cancers [17–21]. Previous studies have shown that the expression of ERRα is significantly up-regulated in colon cancer patients [18]. Additionally, ERRα also promotes cell migration and invasion [22, 23] and controls proliferation and tumourigenic capacity with energy metabolism in colon cancer cells [24]. These findings suggest that ERRα may be a potential biomarker in the progression of colon cancer.

Previous reports reveal that there are some links between the EGFR pathways and ERRα signalling [9, 25, 26]. The MEK/MAPK and PI3K/Akt signalling pathways regulate ERRα transcriptional activity and promote the malignant behaviour of breast cancer cells through increasing ERRα [25], while the overexpression of ERRα also negatively regulates ERK activation [27]. This interaction between ERRα and EGFR suggests a potential novel function of ERRα in the EGF-mediated survival and proliferation of colon cancer cells. Thus, targeting ERRα may be a potential novel therapeutic strategy to enhance the efficiency of EGFR signalling inhibition in colon cancer cells.

In this report, we showed that the suppression of ERRα completely reduced the EGF treatment- induced cell proliferation and survival in colon cancer cells. Furthermore, we found that trametinib partially restrained the up-regulation of ERRα induced by EGF exposure, and the inhibition of ERRα increased the sensitivity of colon cancer cells to trametinib. At last, we combined trametinib with simvastatin, a drug commonly used in the clinic, which has a new reported function of suppressing the transcriptional activity of ERRα [28], and the results showed that this combination synergised to inhibit proliferation and colony formation in vitro as well as the in vivo tumourigenic capacity of colon cancer cells.

## Methods

### Cell lines and culture

The human colon cells that were obtained from the State Key Laboratory of Biotherapy, West China Hospital, Sichuan University included HCT 116 (KRAS G13D), SW480 (KRAS G12 V) and SW1116 (KRASG12A) were grown in Dulbecco’s modified Eagle medium supplemented with 10% foetal bovine serum (FBS, Gibco, USA), 100 mU/mL penicillin, and 100 μg/mL streptomycin in a 5% CO_2_ atmosphere at 37 °C. All the cell lines used were negative for mycoplasma. Trametinib (GSK1120212), XCT790 (HY-10426) and CCCP (HY-100941) were from Medchemexpress. Simvastatin was purchased from J&K Scientific Ltd. (Beijing, China). These agents were all dissolved in dimethyl sulfoxide (DMSO). ERRα luciferase reporter plasmid (pGMERRα-Lu) was purchased from YESEN biology (Shanghai, China). Moreover, the following primary antibodies were obtained from Abcam: UK:rabbit anti-human c-Myc mAb and rabbit anti-human cyclin D1 mAb. The following antibodies were obtained from Santa Cruz: rabbit anti-human Bax mAb, mouse anti-human ERRα mAb, mouse anti-human IDH3A mAb and mouse anti-human GAPDH mAb.

### Tissue samples

The human colon cancer tissue microarrays used in this study were prepared by Shanghai Outdo Biotech Co., Ltd. (Shanghai, China). All the patients signed informed consent forms. This study was approved by the Ethics Committee of Taizhou Hospital of Zhejiang Province.

### Cell viability assay and Clonogenic assay

For the cell proliferation assays, the cells were seeded in 96-well plates for 24 h and were allowed to adhere overnight in regular growth media. After treatment with the indicated drugs, the relative cell growth was measured using the Cell Counting Kit-8 (Dojingdo, Kumamoto, Japan). For the clonogenic assays, the cells were seeded into 35-mm dishes and were cultured in Dulbecco’s modified Eagle medium with 10% foetal bovine serum and 100 IUml-1 penicillin/streptomycin overnight. The cells were then treated with the drug, as indicated, in complete media for 5–6 days. The growth media with or without drug were replaced every 2 days. The remaining cells were fixed with methanol (1%) and formaldehyde (1%), stained with 0.5% crystal violet, and photographed using a digital scanner. All the experiments were performed at least three times. Representative experiments are shown.

### Transfection

The siRNAs against ERRα and the negative controls, the lentiviral shRNA expression vector targeting hERRα and scrambled control (pGPU6/GFP/Neo-shNC) were synthesized by GenePharma (Shanghai, China). ERRα luciferase reporter plasmid (pGMERRα-Lu) was purchased from YESEN biology (Shanghai, China) http://www.yeasen.com/index.htm; The sequence of the shRNA/siRNA/pGMERRα-Lu sense was as follows: pGPU6/GFP/Neo-shERRα#1: 5’-CACCGTGGTGGGCATTGAGCCTCTCTACATTTCAAGAGAATGTAGAGAGGCTCAATGCCCACCATTTTTTG-3′ and pGPU6/GFP/Neo-shERRα#2: 5’-CACCGAATGCACTGGTGTCACATCTGCTGTTCAAGAGACAGCAGATGTGACACCAGTGCATTCTTTTTTG-3′; pGPU6/GFP/Neo-shNC:5'-CACCGTTCTCCGAACGTGTCACGTTTCAAGAGAACGTGACACGTTCGGAGAATTTTTTG-3′; siERRα: 5’-GAAUGCACUGGUGUCACAUCUGCUG-3′. The sequence of ERRα response element (32–91): GGCCTAACTGGCCGGTACCGCT**AGCCTCGATAGCTTGAAGAGGTCACTGTGACCTACAACGAGCTTGAAGAGGTCACTGTGACCTACAACG**GCGCGTAGA [29]; And the siRNAs, shRNAs and pGMERRα-Lu were transfected into the cells using Lipofectamine 2000 (Invitrogen/Life Sciences) according to the manufacturer’s instructions.

### Immunohistochemistry

Immunohistochemistry (IHC) was performed on all colon cancer samples and the tissues of xenograft tumour using biotin-streptavidin HRP detection systems. Paraffin-embedded tissue sections were collected. After deparaffinization with xylene and dehydration in a graded alcohol series, the tissue sections were subjected to antigen retrieval by microwaving in sodium citrate buffer for 10 min and then inhibiting endogenous peroxidase activity. After nonspecific binding was blocked, the slides were incubated with ERRα (1:100) and IDH3A (1:200) antibody (Santa Cruz Biotechnology, CA, USA); c-Myc and Cyclin D1 antibody (1:200; Abcam, Cambridge, UK,) in phosphate-buffered saline (PBS) overnight at 4 °C in a humidified container. Biotinylated secondary antibodies (Zhongshan Golden Bridge Biotechnology Co. Ltd., China) were then used according to the manufacturer’s recommendations. The sections were incubated with HRP-streptavidin conjugates appropriate for detecting ERRα; IDH3A; c-Myc and Cyclin D1. The brown color indicative of peroxidase activity was developed by incubation with 0.1% 3,3-diaminobenzidine (Zhongshan Golden Bridge Biotechnology Co. Ltd. China) in distilled water for 1–3 min at room temperature. The appropriate positive and negative controls were included in each IHC assay.

### Dual luciferase reporter gene assay

A dual luciferase reporter gene assay was performed by using a multifunctional microplate reader (Synergy H1, BioTek, Vermont, USA) and Dual-Luciferase® Report Assay System kit (TransGen Biotech, China). The following procedures were used: Luciferase Reaction buffer II was mixed with thawed Luciferase Reaction substrate II, placed in a centrifuge tube pre-wrapped in foil, and stored at − 80 °C. Thawing took place at room temperature in a dark environment. Stop & Glo buffer was thawed at room temperature and added to 50 x Stop & Glo substrate to prepare a 1 x Stop & Glo Reagent. The cell culture medium was discarded and the cells were washed twice with PBS. Any residual liquid was also removed before 100 uL of 1 x CLB lysis buffer (5 x CLB was diluted to 1× CLB with sterile water) was added into each well. The cells were lysed by shaking on a shaker for 15 min after which 20 uL of the cell lysate was drawn and added to a 96-well opaque detection plate. A total of 100 uL of LARII was the added quickly to the wells containing lysate and gently mixed. Cell lysate was detected on the multi-function microplate reader. Parameters were 10s of reading and 2–3 s delays. The firefly luciferase activity value (F) was measured in relative luminometer units (RFUs). After F was measured, the 96-well plate was immediately taken out of the multifunctional microplate reader and a 100 uL of 1 x Stop & Glo Reagent was added to each well and mixed evenly. The multifunctional microplate reader was used to measure RLUs of renilla luciferase activity (R) over 10s read periods and 2–3 s delays. The relative transcriptional activity of the promoter region was determined by the F/R ratio.

### Transwell chamber migration assay

The cell migration assay was performed using a BD BioCoat™ Matrigel™ Invasion Chamber (BD Biosciences, San Jose, CA. The cells were photographed and counted in three random microscopic fields under a 10× objective to calculate the number of cells that migrated. The graph was plotted for the number of cells that invaded per microscopic field.

### Scratch wounding migration assay

The cell migration ability was assessed by a scratch wound assay. The transfected cells were cultured in 6-well plates. When the cells reached 90% confluence, a scratch wound was created using a pipette tip. The wound edges were photographed with a Nikon Eclipse TE 2000-U (Nikon, Japan), and the scratch widths were analysed using ImageJ software (NIH). Three trials were used for each condition.

### Western blot

The cells were lysed in RIPA buffer (150 mM NaCl, 1% NP-40, 50 mM Tris-HCl, PH 7.4, 1 mM phenylmethylsulfonyl fluoride, 1 μg/ml leupeptin, 1 mM deoxycholic acid and 1 mM EDTA) with protease inhibitors and phosphatase inhibitors (Calbiochem, Darmstadt, Germany). The protein concentration was determined by the Bradford protein assay kit (BioRad). The proteins were separated by SDS-PAGE and were immunoblotted and transferred to polyvinyl difluoride (PVDF) membranes (Millipore) according to standard protocols. Finally, we used the BioRad semidry transfer system to analyse the expression of the proteins, including ERRa, c-Myc, cyclin D1, Bax, and GAPDH.

### Real-time quantitative polymerase chain reaction

The cells were collected in Trizol (Invitrogen, USA) for total RNA extraction as the manufacturer’s protocol instructed. Retrotranscription was performed with the Reverse Transcriptase M-MLV (Takara, Japan). The RT-PCR reactions were performed with a SYBR Premix Ex Taq™ kit (Takara,  Japan) on the iQ5 Real-Time PCR detection system (BioRad, Hercules, USA). The primers used were as follows: ERRa, forward: CACTATGGTGTGGCATCCTGT, reverse: CGTCTCCGCTTGGTGATCTC; IDH3A, forward: AGCCGGTCACCCATCTATGAA, reverse: CytC, forward: CAGTGCCACACCGTTGAAAA reverse: TGCATCGGTTATTTCACACTCC; cyclin D1, forward: GCTTCTGGTGAACAAGCTC, reverse: GTGGGCGTGCAGGCCAGACC; and c-Myc, forward: CAGCTGCTTAGACGCTGGATT, reverse: GTAGAAATACGGCTGCACCGA. The data were analysed using the 2^−ΔΔCT method.

### Cell apoptosis analysis with flow cytometry

A flow-based Annexin V assay was used to measure cell apoptosis after treatment with the drugs. Briefly, the cells were treated with DMSO, trametinib, simvastatin, and trametinib plus simvastatin for 24 h. We used the Annexin V, FITC Apoptosis detection kit (Dojindo Molecular Technologies, Japan) to assess cell apoptosis. The cells were washed in PBS, resuspended in 500 μl of ANX-V binding buffer and were then stained with 5 μl of Annexin-V-fluorescein isothiocyanate (FITC) for 15 min on ice in the dark, according to the manufacturer’s instructions. Subsequent to the staining, the cells were incubated with 10 μl of propidium iodide (PI) for 5 min on ice in the dark. The analyses were performed using a Navios flow cytometer (Beckman Coulter).

### Combination index evaluation

The drug interaction between simvastatin and trametinib was determined by the combination index (CI) value. The CI was evaluated by CompuSyn software (ComboSyn, Inc., Paramus, NJ), using the method proposed by Chou et al. [30]. CI valued < 1, =1, and > 1 indicate synergism, additive and antagonism effects, respectively.

### In vivo xenograft experiment

Female BALB/c nude mice, 4–6 weeks old, were obtained from Dashuo (Chengdu, China). The mice (*n* = 6 per cell line per treatment group) were implanted subcutaneously with HCT116 cells (1.0 × 10^6 cells) in a 100 ul volume using a 23-gauge needle. Each mouse received two subcutaneous injections in the bilateral flank for the development of one tumour. Two weeks after implantation, the mice (n = 6 mice per cell line per treatment group) were assigned to one of four groups including PBS only, trametinib, simvastatin, or a combination of trametinib and simvastatin. The mice were treated daily orally with 1.5 mg/kg trametinib in PBS and/or daily orally with 5 mg/kg simvastatin dissolved in PBS. The tumour diameters were serially measured with a digital calliper (Proinsa, Vitoria, Spain) every 2–3 days, and the tumour volumes were calculated using the following formula: V = (L*W^2)/2, where L and W represent the length and width, respectively.

### Statistical analysis

The data are expressed as the mean ± s.e.m. or the mean ± s.d. Each experiment was conducted at least three times with consistent results. The data were analysed using a two-tailed Student’s t-test by GraphPad Prism 5 (GraphPad Software). Significance is presented as a *P*-value of < 0.05 (*), < 0.01 (**) and < 0.001 (***); non-significant differences are presented as NS.

## Results

### ERRα suppression inhibits the growth of colon cancer cells

**Fig. 1:**
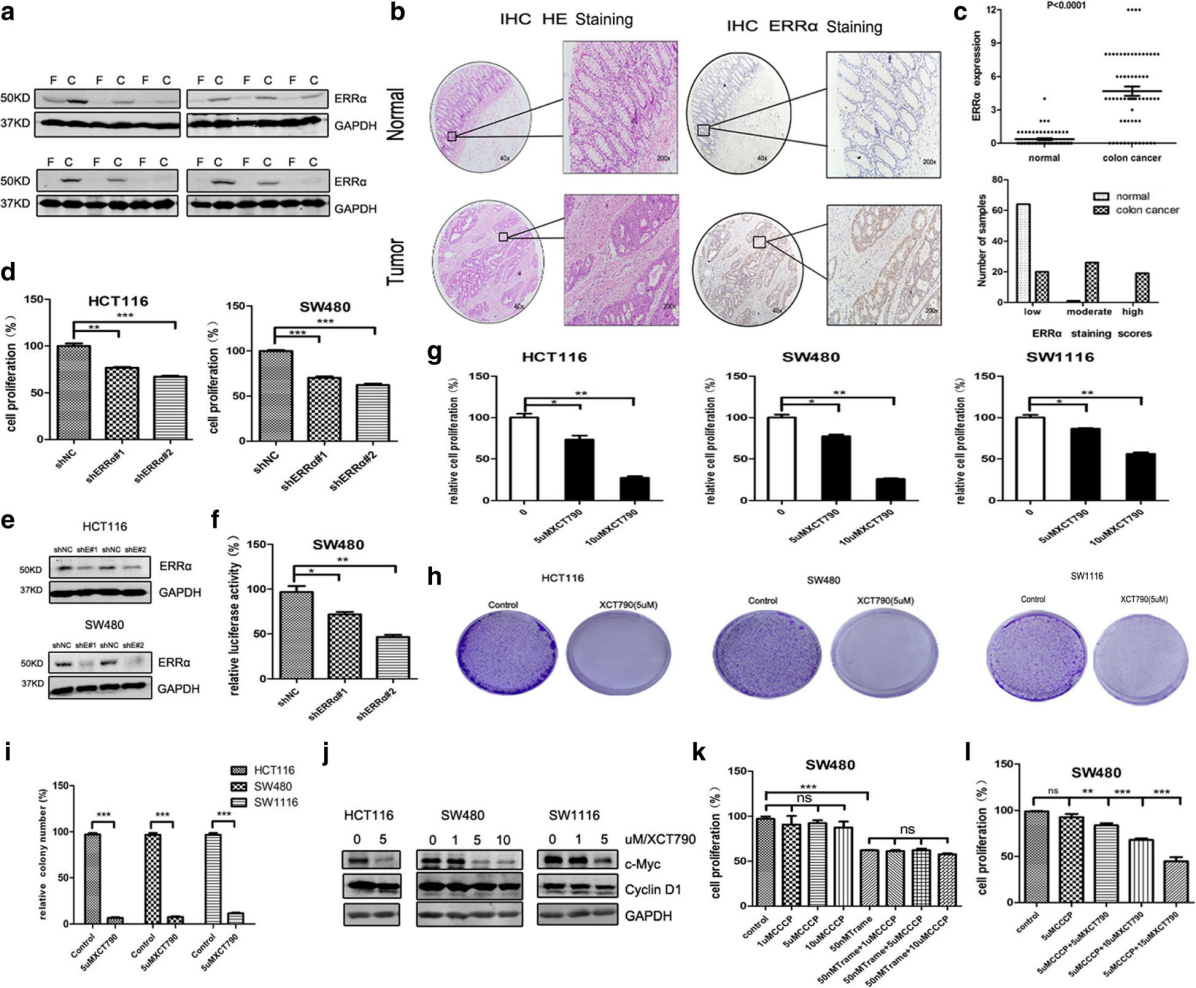
ERRα suppression inhibits the growth of colon cancer cells. **a** ERRα was identified and confirmed by Western blot analysis in 12 pairs of colon cancer tissues (F:distal normal tissues; C:colon cancer tissues). **b** Tissue microarrays were stained with H&E. Representative immunohistochemical staining results for ERRα in human colon tumour tissue and distal normal tissue. **c** The immunoreactivity scores of cancer and distal normal tissue samples are represented by black closed circles. Frequency distribution of ERRα staining scores for tumour tissues and normal tissues (0–3: low expression; 4–7: moderate expression; 8–12: high expression). **d** Cell proliferation assays at day 3 after the HCT116 and SW480 cells were transfected with shERRα#1 and shERRα#2 by using the CCK8. **e** Two individual shRNAs targeting ERRα were introduced into HCT116 and SW480 cells by transfection. Lysates of control and ERRα knockdown HCT116 and SW480 cells were western blotted for ERRα, GAPDH served as a control. **f** In SW480 cell line, the relative luciferase activity of pGMERRα-Lu detected by dual luciferase reporter gene assay decreased in shERRα#1 and shERRα#2 group compared with shNC group. **g** Cell proliferation assays at day 3 after the HCT116, SW480 and SW1116 cells were cultured with XCT790 (5 μM and 10 μM) by using the CCK8. **h, i** Clonogenic assays and qualitative analysis of the HCT116, SW480 and SW1116 cells cultured with 5 μM XCT790 at day 7. **j** The proliferation proteins c-Myc and cyclin D1 were identified and confirmed by Western blot analysis after the colon cancer cells were treated with the indicated concentrations of XCT790 or DMSO for 48 h. **k, l** Cell proliferation assays at day 3 after the SW480 cells were cultured with trametinib (50 nM) or/and CCCP (1uM 5 μM and 10 μM); CCCP (5uM) or/and XCT790 (5 uM 10 μM and 15 μM) by using the CCK8

**Fig. 2:**
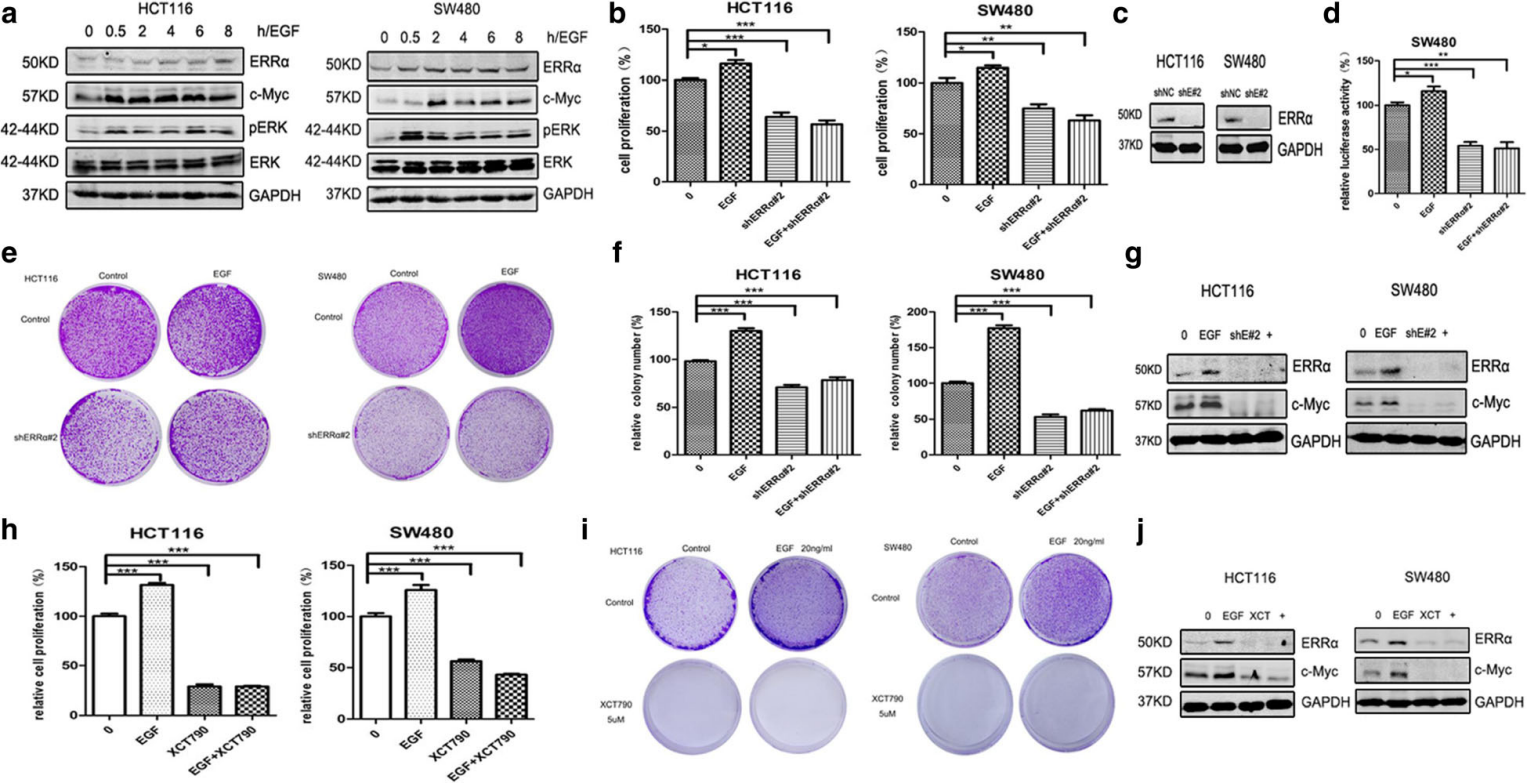
Suppression of ERRα completely reduces the EGF treatment-induced cell proliferation of colon cancer cells. **a** WB for ERRα, c-Myc, cyclin D1, pERK and ERK in the HCT116 and SW480 cells treated with EGF (20/ul) at the indicated times (0.5 h, 2 h, 4 h, 6 h, and 8 h) in serum-free medium. **b** CCK-8 assay for the HCT116 and SW480 cells cultured with shNC or shERRα#2 (or/and 20 ng/μl EGF) for 3 d (* *P*< 0.05; ** *P*< 0.01; *** *P*< 0.001). The data are presented as the mean±SD of the experiments performed in triplicate. **c** The relative expression level of ERRα protein in shERRα#2 group was significantly lower than that in shNC group by WB assay. **d** Dual luciferase reporter gene assay of the SW480 cells treated with shNC or shERRα#2 (or/and 20 ng/μl EGF) in serum-free medium for 48 h. **e, f** Clonogenic assays and qualitative analysis of the HCT116 and SW480 cells cultured with shNC or shERRα#2 (or/and 20 ng/μl EGF) at day 7 (* *P*< 0.05; ** *P*< 0.01; *** P< 0.001). The data are presented as the mean±SD of the experiments performed in triplicate. **g** WB for ERRα and c-Myc in the HCT116 and SW480 cells treated with shNC or shERRα#2 (or/and 20 ng/μl EGF) in serum-free medium for 48 h. **h** CCK-8 assay of the HCT116 and SW480 cells treated with XCT790 (5 μM) (or/and 20 ng/μl EGF) in serum-free medium for 3 d. **i** Clonogenic assays of the HCT116 and SW480 cells cultured with DMSO or 5 μM XCT790 (or/and 20 ng/μl EGF) at day 7 (* *P*< 0.05; ** *P*< 0.01; *** *P*< 0.001). The data are presented as the mean±SD of the experiments performed in triplicate. **j** WB for ERRα and c-Myc in the HCT116 and SW480 cells treated with XCT790 (5 μM) (or/and 20 ng/μl EGF) in serum-free medium for 48 h

To investigate the expression of ERRα in colon cancer tissues, we randomly selected 12 pairs of colon cancer tissue samples for western blot analysis. The results showed that the expression of ERRα was higher in the carcinomatous tissues than in the distal normal tissues (Fig. 1a). Next, we detected ERRα expression by IHC from the pathological tissues of 66 colon cancer patients who had undergone tumour resection. As indicated in Fig. 1b, the expression levels of ERRα were significantly higher in colon tumour tissues than that in distal normal tissues. Unlike in normal tissues, colon tumour tissues also showed positive staining for ERRα in the nucleus. In the normal and cancer tissues, the mean immunoreactivity scores were 0.363 and 4.867 respectively. For ERRα, most of the tumour tissues immunoreactivity scores were 4–7(moderately staining) and 8–12 (highly staining) and the percentages are 41 and 29%, respectively, while majority of the adjacent normal tissues had a score of 0–3 (lowly staining), and the percentage is 98% (Fig. 1c). Then, we also studied the effect of ERRα suppression on the malignant phenotypes of colon cancer cells. The results revealed that the cellular growth and colony information were strongly inhibited in the HCT116 and SW480 cells with shERRα#1 or shERRα#2 transfection compared with the cells transfected with the control shRNA (Figs. 1d-e, 2e-f). To verify whether shERRα performs its inhibition function properly, we constructed a luciferase assay reporter system by transfected the ERRα luciferase reporter plasmid into SW480 cells. The luciferase activity of pGMERRα-Lu significantly decreased in the cells transfected with the shERRα#1 and shERRα#2 (Fig. 1f). We also evaluated whether the inhibition of ERRα activity by a ligand modulated cell proliferation and colony formation in colon cancer cells. XCT790, a potent and specific inverse agonist of ERRα, was used in this further study. The CCK8 assay showed that XCT790 treatment dramatically inhibited colon cell growth (Fig. 1g) and colony formation (Fig. 1h-i). A western blot analysis was used to test the effect of XCT790 treatment on the protein level of ERRα. As expected, colon cancer cells treated with XCT790 showed a reduced level of ERRα compared to the vector control (Additional file 1: Figure S1a). Moreover, XCT790 treatment decreased the expression of genes encoding hyperplasia proteins, including c-Myc and cyclin D1 (Fig. 1j). Then, we also found that the colon cancer cells transfected with si-ERRα displayed less migrated cells compared to the vector control in the transwell assay and wound healing assay (Additional file 1: Figure S1d-g). Collectively, the results suggest that ERRα is involved in the regulation of proliferation and migration of colon cancer cells and plays a role as an oncogene in colon cancer.

### Suppression of ERRα completely reduces the EGF-induced cell proliferation and survival of colon cancer cells

EGFR plays a critical role in the regulation of cell proliferation and differentiation, and EGF is a crucial ligand of EGFR [31]. Here, we found that EGF up-regulated the expressions of ERRα, p-ERK and c-Myc in the HCT116, SW480 and SW1116 cell lines by western blot analysis (Fig. 2a, Additional file 2: Figure S2a). The functional studies revealed that the activated EGFR signalling also promoted cell proliferation, as demonstrated by the Cell Counting Kit-8 assay and colony formation. Further studies indicated that the inhibition of ERRα by shERRα#2, si-ERRα or XCT790 completely reversed the EGF treatment-induced cell proliferation (Fig. 2b-c, e-f, h-i, Additional file 2: Figure S2b) and the expressions of ERRα and c-Myc (Fig. 2g, j, Additional file 2: Figure S2c). In addition, qPCR analysis indicated that shERRα entirely reversed the up-regulation of ERRα, IDH3A [28] and CytC (ERRα’s downstream target) [32] induced by EGF treatment (Additional file 3: Figure S6a), and the up-regulation of luciferase activity of pGMERRα-Lu induced by EGF treatment was also significantly reversed in SW480 cells transfected with the shERRα#2 (Fig. 2d). Together, the above-mentioned data indicated that activated EGFR signalling acts by increasing ERRα to promote the proliferation and survival of colon cancer cells.

### Suppression of ERRα enhances the antitumour property of trametinib in colon cancer cells

**Fig. 3:**
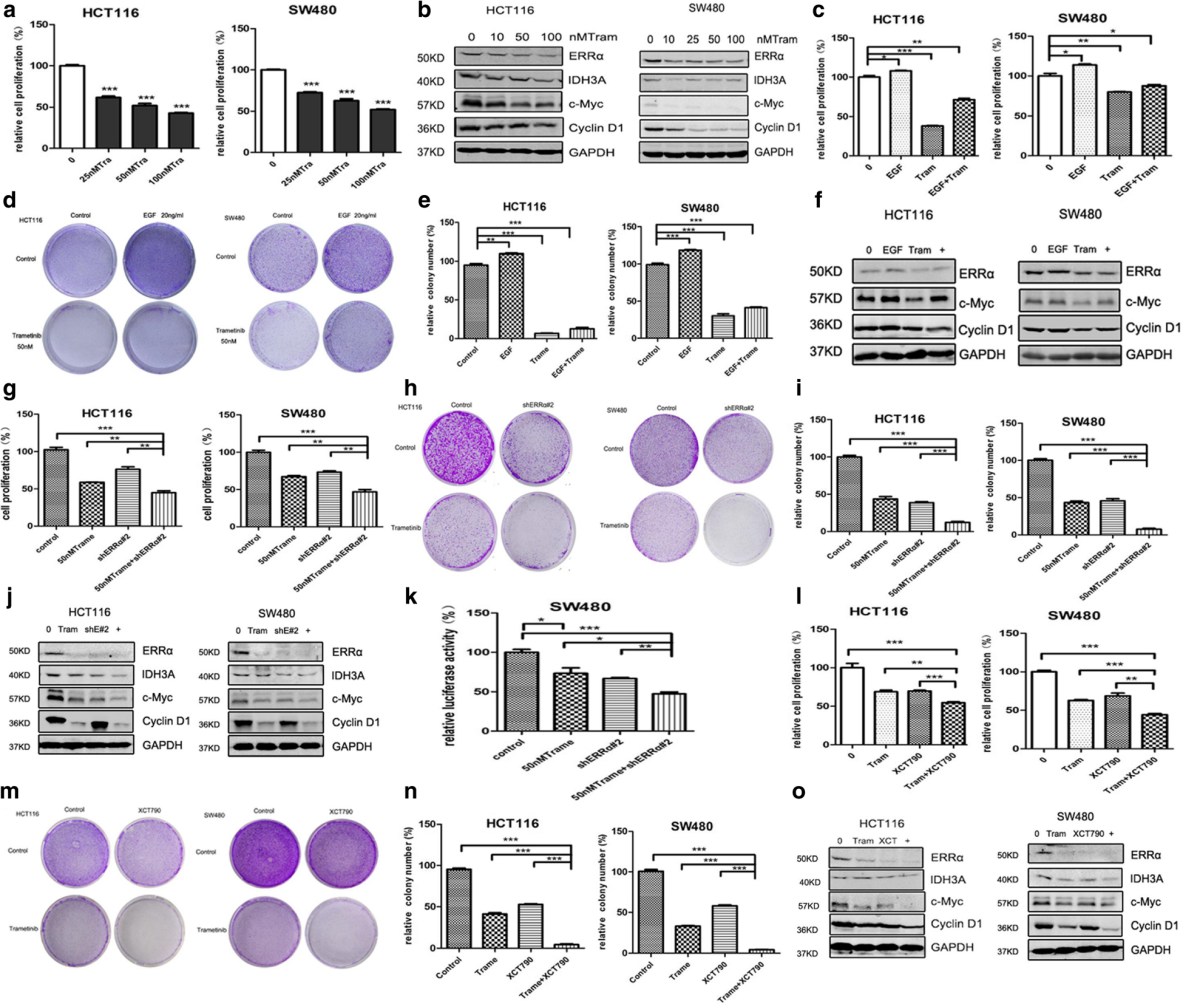
Suppression of ERRα enhances the antitumour property of trametinib in colon cancer cells. **a** Cell proliferation was measured using the Cell Counting Kit-8 (CCK-8) assay in the HCT116 and SW480 cells treated with trametinib at 25 nm, 50 nM and 100 nM for 3 d. **b** WB for ERRα, IDH3A, c-Myc and Cyclin D1 in the HCT116 and SW480 cells treated with the indicated concentrations of trametinib (0–100 nM) or DMSO for 48 h. **c** CCK-8 assay for the HCT116 and SW480 cells treated with DMSO or trametinib (10 nM) (or/and 20 ng/μl EGF) for 3 d. **d, e** Clonogenic assays and qualitative analysis of the HCT116 and SW480 cells cultured with DMSO or 10 nM trametinib (or/and 20 ng/μl EGF) at day 7 (* *P*< 0.05; ** *P*< 0.01; *** *P*< 0.001). The data are presented as the mean±SD of the experiments performed in triplicate. **f** WB for ERRα, c-Myc and Cyclin D1 in the HCT116 and SW480 cells treated with the DMSO or 10 nM trametinib for 48 h (or/and 20 ng/μl EGF) for 2 d. **g** CCK-8 assay for the HCT116 and SW480 cells treated with shERRα#2 (or/and 50 nM trametinib) for 3 d**. h, i** Clonogenic assays and qualitative analysis of the HCT116 and SW480 cells cultured with DMSO or 50 nM trametinib (or/and shERRα#2) at day 7. **j** WB for ERRα, IDH3A, c-Myc and Cyclin D1 in the HCT116 and SW480 cells treated with shERRα#2 (or/and 50 nM trametinib) for 2 d. **k** Dual luciferase reporter gene assay of the SW480 cells treated with shNC or shERRα#2 (or/and 50 nM trametinib) for 48 h. **l** CCK-8 assay for the HCT116 and SW480 cells treated with 50 nM trametinib and 5 μM XCT790 for 3 d. **m, n** Clonogenic assays and qualitative analysis of the HCT116 and SW480 cells cultured with DMSO or 50 nM trametinib (or/and 5 μM XCT790) at day 7. **o** WB for ERRα, IDH3A, c-Myc and Cyclin D1 in the HCT116 and SW480 cells cultured with DMSO or 50 nM trametinib (or/and 5 μM XCT790) for 48 h

RAF-MEK-ERK (mitogen-activated protein kinase (MAPK) pathway) signalling is frequently activated in human cancers, resulting in an increase in cellular proliferation [23]. Trametinib, a specific MEK inhibitor, is clinically used in melanoma (Additional file 2: Figure S2d). Here, we found that trametinib inhibited cell growth (Fig. 3a) and decreased the expression of ERRα and its downstream target IDH3A (Fig. 3b). Further investigations indicated that trametinib partially reversed the elevated cell number and colony formation induced by EGF stimulation (Fig. 3c-e). QPCR analysis indicated that trametinib did not entirely reverse the up-regulation of ERRα, IDH3A and CytC induced by EGF treatment compared with the trametinib treatment alone (Additional file 3: Figure S6b), and western blot analysis also demonstrated that trametinib did not entirely reverse the up-regulation of ERRα, c-Myc and cyclin D1 induced by EGF treatment (Fig. 3f, Additional file 2: Figure S2e).

Therefore, we combined trametinib with shERRα, si-ERRα and XCT790 to investigate whether ERRα suppression enhances the cytotoxicity of trametinib against colon cancer. Our results showed that the combination was more effective at restraining cell proliferation (Fig. 3g, l, Additional file 2: Figure S2f) and colony formation (Fig. 3h-i, m-n). And the luciferase activity of pGMERRα-Lu was more significantly decreased in SW480 cells treated by combined trametinib and shERRα#2 (Fig. 3k). Western blot analysis also demonstrated that the combination inhibited ERRα, IDH3A, c-Myc and cyclin D1 more thoroughly compared to the single treatment (Fig. 3j, o, Additional file 2: Figure S2g-i). Furthermore, qPCR analyses also showed a substantial reduction in the ERRα and its downstream target genes IDH3A and CytC in the combination group (Additional file 3: Figure S6c). Although trametinib is an effective drug that suppresses the growth of colon cancer cells, it did not achieve adequate cytotoxicity and inhibit the over-expression of ERRα induced by EGF. This implies that the co-inhibition of ERRα and MEK achieved more efficiency.

### Simvastatin decreases the transcriptional activity of ERRα in colon cancer cells

**Fig. 4:**
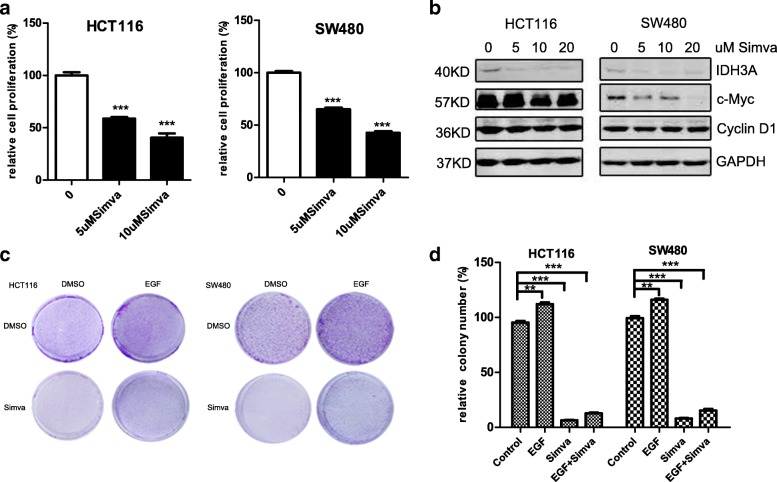
Simvastatin decreases the transcriptional activity of ERRα in colon cancer cells. **a** Cell proliferation assays at day 3 for the HCT116 and SW480 cells cultured with simvastatin (5 μM and 10 μM) using the Cell Counting Kit-8. **b** WB for IDH3A, c-Myc and cyclin D1 in the HCT116 and SW480 cells treated with the indicated concentrations of simvastatin (0–20 μM) or DMSO for 48 h. **c, d** Clonogenic assays and qualitative analysis of the HCT116 and SW480 cells cultured with DMSO or 5 μM simvastatin (or/and 20 ng/μl EGF) at day 7

To determine whether FDA-approved inhibitors exist that block the activity of ERRα, we performed a literature review and found that statins and bisphosphonates inhibit the activity of ERRα by blocking its cholesterol modification. Cholesterol is identified as the first functional endogenous ERRα ligand, and it increases the transcriptional activity of ERRα, while statins lower intracellular sterol levels, thus attenuating ERRα transactivation [28]. In addition, we found that simvastatin decreased the expression of its downstream target IDH3A and proliferation-related genes, such as c-Myc and cyclin D1, in the HCT116 and SW480 cell lines (Fig. 4b). The functional studies revealed that simvastatin inhibited the proliferation and colony formation of colon cancer cells (Fig. 4a, c-d, Additional file 4: Figure S3a-c). Moreover, consistent with the effect of trametinib, simvastatin partially reversed the EGF treatment-induced proliferation (Fig. 4c-d, Additional file 4: Figure S3b-c). Taken together, our results indicated that simvastatin decreased the transcriptional activity of ERRα and inhibited tumour growth in colon cancer.

### Anti-tumour effect of a combination of trametinib and simvastatin

**Fig. 5:**
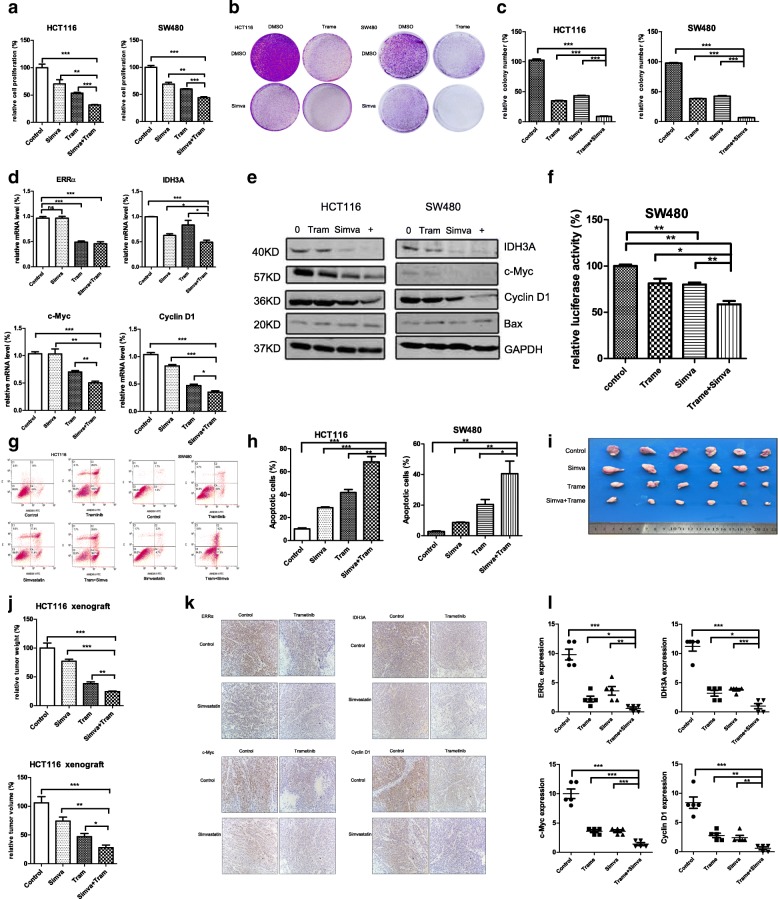
Antitumour effect of the combination of trametinib and simvastatin. **a** Cell proliferation assays at day 3 for the HCT116 and SW480 cells cultured with simvastatin (10 μM) or DMSO in the presence or absence of 50 nM trametinib. **b, c** Clonogenic assays and qualitative analysis of the HCT116 and SW480 cells cultured with DMSO or 10 μM simvastatin (or/and 50 nM trametinib) at day 7. **d** Quantitative real-time PCR analysis of ERRα and IDH3A, c-Myc, cyclin D1 in the HCT116 cells treated with 10 μM simvastatin (or/and 50 nM trametinib) for 48 h. GAPDH was used as a control. **e** WB for IDH3A, c-Myc, cyclin D1 and Bax in the HCT116 and SW480 cells treated with 10 μM simvastatin (or/and 50 nM trametinib) for 48 h. **f** Dual luciferase reporter gene assay of the SW480 cells treated with 10 μM simvastatin (or/and 50 nM trametinib) for 48 h. **g, h** Flow cytometric analysis of the cell cycle of the HCT116 and SW480 cells treated with DMSO or 10 μM simvastatin (or/and 50 nM trametinib) for 48 h. **i, j** Tumour formation assays in the nude mice subcutaneously injected with HCT116 cells (1× 10^6). When the tumours reached 3 mm in diameter, the mice were orally with DMSO, simvastatin (30 mg/kg) or/and trametinib (1.5 mg/kg) daily. The tumour sizes were measured after 2 weeks. The graph shows the relative tumour volume and weight of each group (*n*=6 animals for each group). The data presented as the mean±SD, *n*=3 independent experiments. *P < 0.05, **P < 0.01, ***P < 0.001 using Student’s t test (two-tailed). **k** Representative immunohistochemical staining results for ERRα, IDH3A, c-Myc and Cyclin D1 in xenograft tumour tissues. **l** The graph shows the immunoreactivity scores of ERRα, IDH3A, c-Myc and Cyclin D1 in each group (n=6 animals for each group)

The above-mentioned data indicated that simvastatin might strengthen the antitumour efficiency of trametinib by inhibiting the activity of ERRα. Further CCK8 assays indicated that simvastatin significantly enhanced the cytotoxicity of trametinib in the HCT 116 and SW480 cells (Fig. 5a). The colony formation assays revealed that simvastatin, combined with trametinib, inhibited cell survival more significantly than simvastatin or trametinib alone in the two colon cancer cell lines (Fig. 5b-c). Moreover, the flow cytometry assays showed that this combination also produced a combined activity, with regard to cell apoptosis in colon cancer cells (Fig. 5g-h). Western blot analysis demonstrated that simvastatin synergised with trametinib and dramatically reduced the expression of the IDH3A, the proliferation-related genes c-Myc and cyclin D1, and incresesd the pro-apoptotic gene Bax (Fig. 5e). Next, quantitative real-time PCR showed that the combination therapy strongly decreased the mRNA expression of ERRα and its downstream targets IDH3A, c-Myc, and cyclin D1 compared with the single drug in the HCT116 cells (Fig. 5d), and the similar results were also found in SW480 cells (Additional file 3: Figure S6d). Furthermore, the luciferase activity of pGMERRα-Lu was more potently decreased in SW480 cells when combined trametinib and simvastatin (Fig. 5f). To investigate the combined effects, we calculated the combination index (CI) values and the Fa values using CompuSyn software (ComboSyn, Inc., Paramus, NJ, USA). According to the method proposed by Chou et al., the combination index (CI) values of < 1, =1, > 1, indicate synergistic, additive and antagonistic effects, respectively[30] . The combination index (CI) values were 0.03 and 0.19 in the HCT116 and sw480 cells, respectively, indicating that the combined therapy produced a synergistic effect in the two cell lines (Additional file 5: Figure S4a).

To investigate the combined effect in vivo, we implanted HCT116 tumours in nude mice, and they were assigned to the following four groups: untreated control, trametinib, simvastatin, or a combination of trametinib and simvastatin. The combination group showed a statistically significant reduction in tumour volume and weight compared with the vehicle-treated controls or the monotherapy groups in the HCT116 xenografts (Fig. 5i-j). Next, we detected ERRα, IDH3A, c-Myc and Cyclin D1 expression by immunostaining pathological tissue sections of xenograft tumour. As indicated in Fig. 5k-l, the overall protein expression levels of ERRα, IDH3A, c-Myc and Cyclin D1 were significantly weaker in combination group. Furthermore, a western blot was preformed to investigate the expression of proliferative proteins in the lysate from the xenografts. In contrast to the monotherapy groups, a combination of trametinib and simvastatin significantly down-regulated the expressions of c-Myc and cyclin D1 (Additional file 5: Figure S4b). Altogether, our findings unveiled that trametinib, combined with simvastatin, produced synthetic lethality in vitro and in vivo.

## Discussion

ERRα regulates multiple biosynthetic pathways involved in energy metabolism [15, 33]. Recently, increasing evidence supports a critical role for ERRα as a pro-tumourigenic factor, and the vast majority of studies show that high ERRα expression is correlated with a poor clinical outcome in endocrine-related cancers [19, 34, 35]. In colon cancer, ERRα expression is significantly up-regulated compared with adjacent normal colon tissues [18]. Notably, we verified a new insight into the pro-tumourigenic function of ERRα in colon cancer. In our study, shERRα and XCT790 (which acts as a superagonist of ERRα) were used to suppress the expression of ERRα. The results showed that ERRα was required for colon cancer cell growth in vitro, and silencing ERRα decreased the migration ability of the HCT116, SW480 and SW1116 cell lines, which was consistent with a previous study [22, 24]. Otherwise, XCT 790 is also a potent, fast-acting, mitochondrial uncoupler independent of its inhibition function of ERRα [36]. To explore whether XCT790 inhibits the cell growth and proliferation mainly by inhibiting ERRα activity, but independent of its disruption on the mitochondrial transmembrane electrochemical gradients. We used CCCP, a chemical mitochondrial uncoupler that could inhibit the mitochondrial respiration in our study [36], and found CCCP could not effectively suppress cell growth when taken alone, and combined with trametinib also has no synergistic effect on cell growth (Fig. 1k, Additional file 1: Figure S1b). And under the suppression of the mitochondrial respiration by CCCP, XCT790 could still significantly inhibit colon cancer cells growth (Fig. 1l, Additional file 1: Figure S1c), suggesting that XCT790 mainly acts through inhibiting ERRα activity to suppress cell growth and proliferation. Importantly, these effects are completely independent of its function of disrupting mitochondrial transmembrane electrochemical gradients. Furthermore, our study first found that the suppression of ERRα completely reduced the survival of EGF-treated colon cancer cells, though it has been known for many years that ERRα expression is regulated, in part, via the EGF signalling pathway. Thus, our data suggested that ERRα was an oncogene and acted as a novel target for colon cancer therapy. However, all the ERRα antagonists (DES, XCT790 and SR16388) are still in pre-clinical study.

The presence of the oncogenic BRAF/KRAS mutation excludes metastatic colon cancer patients from targeted therapies, leaving them with only chemotherapy or no treatment if the disease is chemorefractory. Additional target drugs to prolong the PFS (progression-free survival) and OS (overall survival) are limited in metastatic colon cancers, suggesting the need to target other pathways. Trametinib is a highly specific and potent MEK1/2 inhibitor that suppresses the activity of RAS/ERK signalling, which is expected to inhibit the growth of cancers with the RAS/BRAF mutation. However, due to drug resistance, trametinib has only been approved by the FDA, in combination with dabrafenib, for the treatment of BRAF-mutated metastatic melanoma and advanced non-small cell lung cancer.

In this study, we found that trametinib down-regulated ERRα gene expression and inhibited its transcriptional activity probably through posttranscriptional regulation, because the immunoblot analysis showed that trametinib rapidly accelerated the degradation rate of ERRα, and it was reversed by MG132 (Additional file 2: Figure S2j-k). Although trametinib is an effective medicine to suppress the growth of colon cancer cells, the expression of the ERRα was not completely suppressed by trametinib in the presence of the EGF. Our data showed that ERRα played a central role in the EGF-mediated growth of colon cancer cells; thus, we hypothesized that inhibiting ERRα may increase the sensitivity of colon cancer cells to trametinib. We combined trametinib and XCT790 or shERRα and found that suppressing ERRα increased the antitumour effect of trametinib. Therefore, a combination of trametinib and XCT790 might be a good choice for colon cancer treatment. However, XCT790 is not approved in clinical applications; thus, we need to find a safe and effective drug combined with trametinib to inhibit ERRα activity entirely.

Simvastatin, an oral lipid-lowering drug, is approved by the FDA. Many studies demonstrate its antitumour activity in several cancer types [37–39]. Recently, cholesterol was identified as an endogenous ERRa agonist, and ERRa transcriptional activity is significantly enhanced by cholesterol and suppressed by statins [28]. Thus, we replaced XCT790 with simvastatin and found that this combination decreased ERRα expression completely and had a synergistic effect, inhibiting proliferation and colony formation in vitro as well as the in vivo tumourigenic capacity of colon cancer cells.

Furthermore, we detected the expression of HMGCR (3-hydroxy-3-Methylglutaryl -coenzyme A) in the tissues, and the results showed that the expression of HMGCR was also higher in carcinomatous tissues than that of the distal normal tissues in 12 pairs of colon cancer tissues (Additional file 6: Figure S5). HMGCR is a key enzyme in the mevalonate pathway in tissues, and its high expression may suggest a high concentration of produced cholesterol and high activities of ERRα. Thus, simvastatin, a HMG-CoA reductase inhibitor, synergised with trametinib, might be a good choice to inhibit the tumourigenic capacity of colon cancer cells.

It is known that various preclinical and therapeutic strategies using trametinib combined with another target drug in BRAF/KRAS mutant colon cancers were developed [40, 41]. However, none of these strategies are approved for clinical use due to safety issues or a lack of objective responses during clinical trials.

## Conclusions

**Fig. 6:**
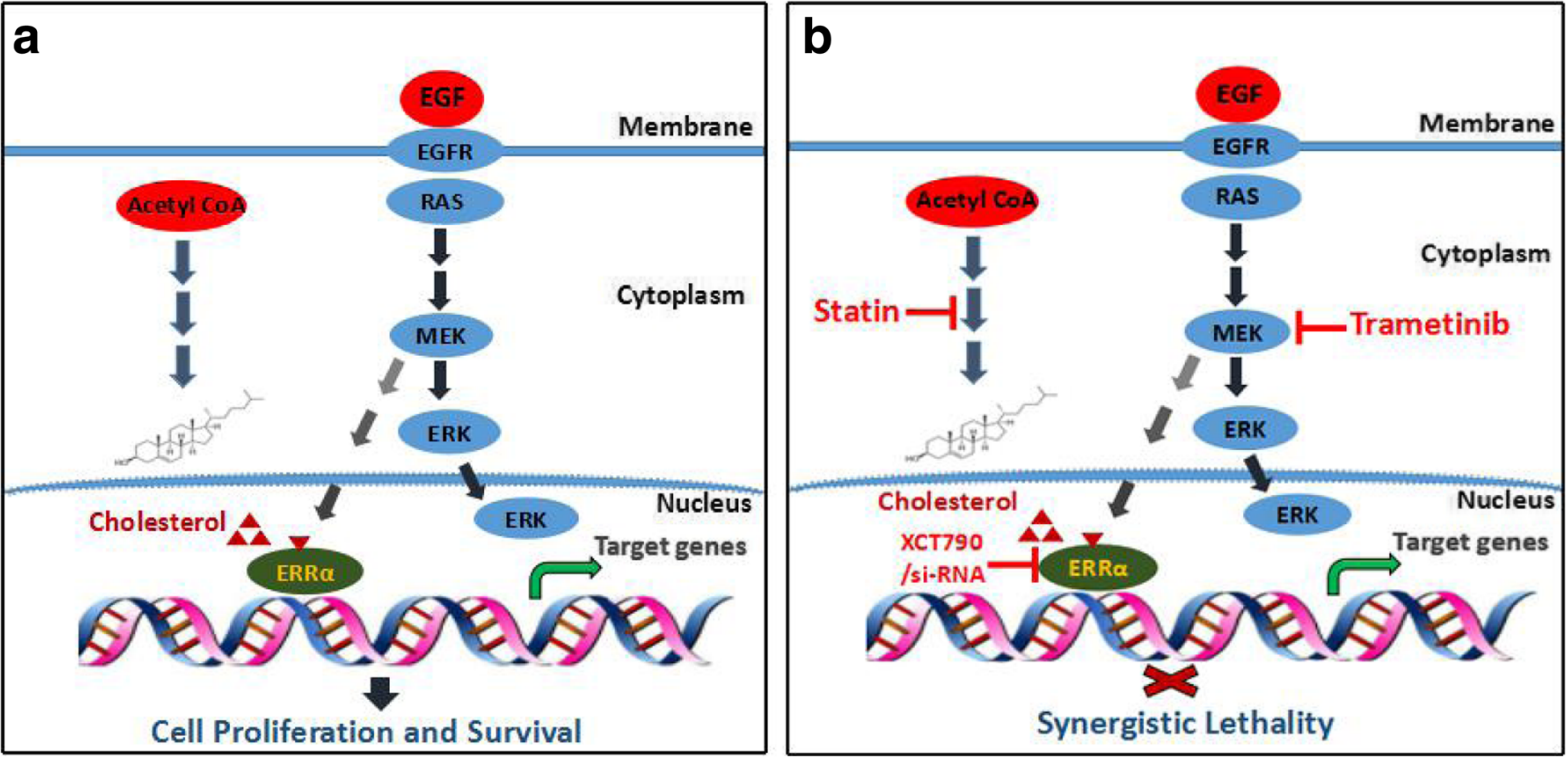
A Schematic diagram of how ERRα mediates the synthetic lethality effects of trametinib and simvastatin. Proposed working model: **a** EGF stimulation promotes ERRα expression, probably via the up-regulation of RAS-ERK signalling. Cholesterol serves as a natural ERRa agonist to increase ERRα transcriptional activity, thereby promoting colon cancer cell proliferation and tumour growth. **b** Trametinib inhibits ERRα expression, perhaps via directly down-regulating RAS-ERK signalling. A reduction in cholesterol synthesis by statins decreases ERRa transcriptional activity; therefore, trametinib combined with simvastatin effectively inhibits colon cancer cell proliferation and tumour growth

In our study, the results of in vitro and in vivo experiments demonstrate that the suppression of ERRα by simvastatin enhances the antitumour properties of trametinib in colon cancer cells. In addition, we provide a novel therapeutic strategy for colon cancer by combining trametinib and simvastatin to inhibit the ERRα signaling axis (Fig. 6a-b).

## Additional files

Additional file 1: **Figure S1.** ERRα controls the growth of colon cancer cells. a ERRα was identified and confirmed by Western blot analysis when colon cancer cells were treated with the indicated concentrations of XCT790 (0–10 μM) or DMSO for 48 h. b Cell proliferation assays at day 3 after the SW480 cells were cultured with trametinib (50 nM) or/and CCCP (1uM 5 μM and 10 μM) by using the Cell Counting Kit-8. c Cell proliferation assays at day 3 after the SW480 cells were cultured with CCCP (5uM) or/and XCT790 (5 uM, 10 μM and 15 μM) by using the Cell Counting Kit-8. d, e Wound distance percentage of the HCT116, SW480 and SW1116 cells at 0, 24 and 48 h after si-ERRα#2 treatment; (* *P*< 0.05: ** *P*< 0.01; *** *P*< 0.001). The data are presented as the mean±SD of the experiments performed in triplicate. f, g Invasion assay of the HCT116, SW480 and SW1116 cells after 24 and 48 h of transfection with si-ERRα#2 (* P< 0.05: ** P< 0.01; *** P< 0.001). The data are presented as the mean±SD of the experiments performed in triplicate. (PDF 898 kb)

Additional file 2: **Figure S2.** Suppression of ERRα completely reduces the EGF treatment-induced cell proliferation and enhances the cytotoxicity of trametinib. a WB for ERRα, c-Myc, cyclin D1, pERK and ERK in the SW1116 cells treated with EGF (20/μl) at the indicated times (0.5, 2, 4, 6 and 8 h) in serum-free medium. b CCK-8 assay for the HCT116 and SW480 cells cultured with si-NC or si-ERRα (or/and 20 ng/μl EGF) for 3 d (* *P*< 0.05; ** *P*< 0.01; *** *P*< 0.001). The data are presented as the mean±SD of the experiments performed in triplicate. c WB for ERRα, c-Myc and cyclin D1 in the HCT116 and SW480 cells treated with si-NC or si-ERRα (or/and 20 ng/μl EGF) in serum-free medium for 48 h. d WB for pERK and ERK in the HCT116, SW480 and SW1116 cells treated with the indicated concentrations of trametinib (0–100 nM) or DMSO for 48 h. e WB for ERRα, c-Myc and cyclin D1 in the SW1116 cells treated with DMSO or 10 nM trametinib (or/and 20 ng/μl EGF) for 48 h. f CCK-8 assay for the HCT116 and SW480 cells treated with si-ERRα (or/and 50 nM trametinib) for 3 d. g, h, i WB for ERRα, IDH3A, c-Myc and Cyclin D1 in the HCT116, SW480 and SW1116 cells treated with si-ERRα (or/and 50 nM trametinib) for 2 d. j WB for ERRα in the HCT116 and SW1116 cells treated with cycloheximide (10 μg/ml) or trametinib combined with cycloheximide in a time-course experiment. k WB for ERRα in the HCT116 and SW1116 treated with trametinib (50 nM, 48 h) or DMSO supplemented with or without MG132 (10 μM) for 8 h. (PDF 898 kb)

Additional file 3: **Figure S6.** Quantitative RT–PCR analysis for interactions between drugs. **a** Quantitative real-time PCR analysis of ERRα, IDH3A and CytC in the SW480 cells treated with shNC or shERRα#2 (or/and 20 ng/μl EGF) for 2 d. **b** QPCR for ERRα, IDH3A and CytC in the SW480 cells treated with DMSO or trametinib (10 nM) (or/and 20 ng/μl EGF) for 2 d. **c** SW480 cells cultured with DMSO or 50 nM trametinib (or/and shERRα#2) at day 2 by qPCR assay. **d** Quantitative real-time PCR analysis of ERRα, IDH3A and CytC in the SW480 cells treated with 10 μM simvastatin (or/and 50 nM trametinib) for 2d. (PDF 949 kb)

Additional file 4: **Figure S3.** Simvastatin decreases the transcriptional activity of ERRα in colon cancer cells. a Cell proliferation assays at day 3 for the SW1116 cells cultured with simvastatin (5 μM and 10 μM) using the Cell Counting Kit-8. b Clonogenic assays and qualitative analysis of the SW1116 cells cultured with DMSO or 5 μM simvastatin (or/and 20 ng/μl EGF) at day 7. (PDF 947 kb)

Additional file 5: **Figure S4.** Antitumour effect of the combination of trametinib and simvastatin. a The CI and Fa of the HCT116 and SW480 cells treated with DMSO or 10 μM simvastatin (or/and 50 nM trametinib) for 48 h by CompuSyn software. b The tumours were analysed for proliferation (c-Myc, cyclin D1) and proapoptotic (Bax) proteins by an immunoblot assay. GAPDH was used as the protein loading control. (PDF 947 kb)

Additional file 6: **Figure S5.** a HMGCR is higher in carcinomatous tissues. HMGCR were identified and confirmed by Western blot analysis in 12 pairs of colon cancer tissues. (F:distal normal tissues;C:colon cancer tissues). (PDF 946 kb)

## Abbreviations

AKT: Protein kinase B
CytC: Cytochrome c
EGF: Epidermal growth factor
EGFR: Epidermal growth factor receptor
ERRα: Oestrogen-related receptor α
FDA: Food and Drug Administration
HMGCR: 3-hydroxy-3-Methylglutaryl-coenzyme A
IDH3A: Isocitrate Dehydrogenase 3 (NAD(+)) Alpha
MAPK: Extracellular signal regulated kinase
MEK: Mitogen-activated protein kinase
MG132: Proteasome inhibitor MG132
OS: Overall survival
PFS: Progression-free survival
PI3K: Phosphoinositide 3-kinase
RAS: Rat sarcoma
siRNA: Short interfering ribonucleic acid
