# Editorial expression of concern: ERRα suppression enhances the cytotoxicity of the MEK inhibitor trametinib against colon cancer cells

**DOI:** 10.1186/s13046-025-03507-3

**Published:** 2025-08-15

**Authors:** Sheng Zhou, Hongwei Xia, Huanji Xu, Qiulin Tang, Yongzhan Nie, Qi Yong Gong, Feng Bi

**Affiliations:** 1 Department of Medical Oncology, Cancer Center, West China Hospital, Sichuan University, Chengdu, Sichuan Province China; 2 Laboratory of Molecular Targeted Therapy in Oncology, State Key Laboratory of Biotherapy and Cancer Center, West China Hospital, Sichuan University, Chengdu, Sichuan Province China; 3 Collaborative Innovation Center for Biotherapy, Chengdu, Sichuan Province China; 4 State Key Laboratory of Cancer Biology & Xijing Hospital of Digest Diseases, Fourth Military Medical University, Xi’an, Shanxi Province China; 5 Department of Radiology, West China Hospital, Sichuan University, Chengdu, Sichuan Province China; 6 Laboratory of Molecular Targeted Therapy in Oncology, Department of Medical Oncology, West China Hospital, Sichuan University, Chengdu, 610041 Sichuan Province China

**Correction:** ***J Exp Clin Cancer Res*** **37, 218 (2018)**

10.1186/s13046-018-0862-8; Published: 5 September 2018 

The Editor-in-Chief would like to alert the readers that concerns have been raised regarding some of the western blot data presented in this article. Specifically, Fig. 3j HCT116 c-Myc lanes 3 and 4 and SW480 c-Myc lanes 1 and 2 appear highly similar. In addition, Figs. 2a (pERK), 3j (c-Myc) and Supplementary Fig. S2a (ERK and pERK) of this article appear highly similar to Figs. 3a and 4a of the authors’ later publication [1].

The authors have stated that the blots were inadvertently reused due to the experiments for the two studies being performed simultaneously, and the reused data referred to the same cell lines and conditions. The authors have also performed validation experiments, but have been unable to provide the original raw data for Fig. 3j c-Myc. Readers are therefore advised to interprent these results with caution.

Sheng Zhou, Hongwei Xia, Huanji Xu, Qiulin Tang and Feng Bi agree with this Editorial Expression of Concern, but not the wording of the concerns. Yongzhan Nie and Qi yong Gong did not respond to any correspondence from the Publisher about this Editorial Expression of Concern.
