# Supplementary material for: ERRα suppression enhances the cytotoxicity of the MEK inhibitor trametinib against colon cancer cells
Source: J Exp Clin Cancer Res. 2018 Sep 5;37:218. doi: 10.1186/s13046-018-0862-8 (PMC6125878; doi:10.1186/s13046-018-0862-8)
Supplement: Supplementary file 1 — Figure S1. ERRα controls the growth of colon cancer cells. a ERRα was identified and confirmed by Western blot analysis when colon cancer cells were treated with the indicated concentrations of XCT790 (0–10 μM) or DMSO for 48 h. b Cell proliferation assays at day 3 after the SW480 cells were cultured with trametinib (50 nM) or/and CCCP (1uM 5 μM and 10 μM) by using the Cell Counting Kit-8. c Cell proliferation assays at day 3 after the SW480 cells were cultured with CCCP (5uM) or/and XCT790 (5 uM, 10 μM and 15 μM) by using the Cell Counting Kit-8. d, e Wound distance percentage of the HCT116, SW480 and SW1116 cells at 0, 24 and 48 h after si-ERRα#2 treatment; (* P< 0.05: ** P< 0.01; *** P< 0.001). The data are presented as the mean±SD of the experiments performed in triplicate. f, g Invasion assay of the HCT116, SW480 and SW1116 cells after 24 and 48 h of transfection with si-ERRα#2 (* P< 0.05: ** P< 0.01; *** P< 0.001). The data are presented as the mean±SD of the experiments performed in triplicate. (PDF 898 kb) [file 13046_2018_862_MOESM1_ESM.pdf]

Additional file 1:

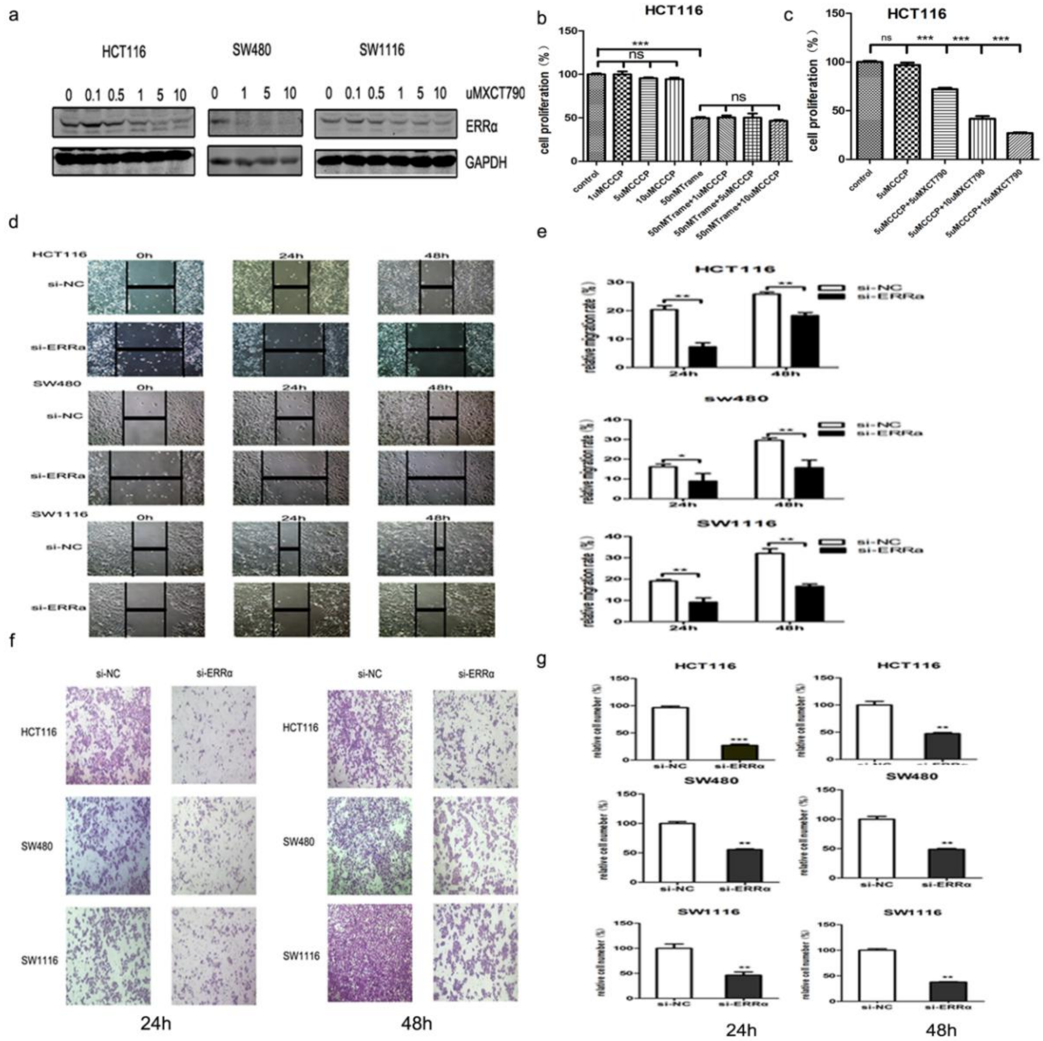

Figure S1. ERRα controls the growth of colon cancer cells. **a** ERRα was identified and confirmed by Western blot analysis when colon cancer cells were treated with the indicated concentrations of XCT790 (0-10 μM) or DMSO for 48 h. **b** Cell proliferation assays at day 3 after the SW480 cells were cultured with trametinib (50nM) or/and CCCP (1uM 5 μM and 10 μM) by using the Cell Counting Kit-8. **c** Cell proliferation assays at day 3 after the SW480 cells were cultured with CCCP (5uM) or/and XCT790 (5 uM, 10 μM and 15 μM) by using the Cell Counting Kit-8.

d, e Wound distance percentage of the HCT116, SW480 and SW1116 cells at 0, 24 h, and 48 h after si-ERRα#2 treatment; (* P<0.05: ** P<0.01; *** P<0.001). The data are presented as the mean±SD of the experiments performed in triplicate. **f, g** Invasion assay of the HCT116, SW480 and SW1116 cells after 24 h and 48 h of transfection with si-ERRα#2 (* P<0.05: ** P<0.01; *** P<0.001). The data are presented as the mean±SD of the experiments performed in triplicate.
