# Supplementary material for: ERRα suppression enhances the cytotoxicity of the MEK inhibitor trametinib against colon cancer cells
Source: J Exp Clin Cancer Res. 2018 Sep 5;37:218. doi: 10.1186/s13046-018-0862-8 (PMC6125878; doi:10.1186/s13046-018-0862-8)
Supplement: Supplementary file 2 — Figure S2. Suppression of ERRα completely reduces the EGF treatment-induced cell proliferation and enhances the cytotoxicity of trametinib. a WB for ERRα, c-Myc, cyclin D1, pERK and ERK in the SW1116 cells treated with EGF (20/μl) at the indicated times (0.5, 2, 4, 6 and 8 h) in serum-free medium. b CCK-8 assay for the HCT116 and SW480 cells cultured with si-NC or si-ERRα (or/and 20 ng/μl EGF) for 3 d (* P< 0.05; ** P< 0.01; *** P< 0.001). The data are presented as the mean±SD of the experiments performed in triplicate. c WB for ERRα, c-Myc and cyclin D1 in the HCT116 and SW480 cells treated with si-NC or si-ERRα (or/and 20 ng/μl EGF) in serum-free medium for 48 h. d WB for pERK and ERK in the HCT116, SW480 and SW1116 cells treated with the indicated concentrations of trametinib (0–100 nM) or DMSO for 48 h. e WB for ERRα, c-Myc and cyclin D1 in the SW1116 cells treated with DMSO or 10 nM trametinib (or/and 20 ng/μl EGF) for 48 h. f CCK-8 assay for the HCT116 and SW480 cells treated with si-ERRα (or/and 50 nM trametinib) for 3 d. g, h, i WB for ERRα, IDH3A, c-Myc and Cyclin D1 in the HCT116, SW480 and SW1116 cells treated with si-ERRα (or/and 50 nM trametinib) for 2 d. j WB for ERRα in the HCT116 and SW1116 cells treated with cycloheximide (10 μg/ml) or trametinib combined with cycloheximide in a time-course experiment. k WB for ERRα in the HCT116 and SW1116 treated with trametinib (50 nM, 48 h) or DMSO supplemented with or without MG132 (10 μM) for 8 h. (PDF 898 kb) [file 13046_2018_862_MOESM2_ESM.pdf]

Additional file 2:

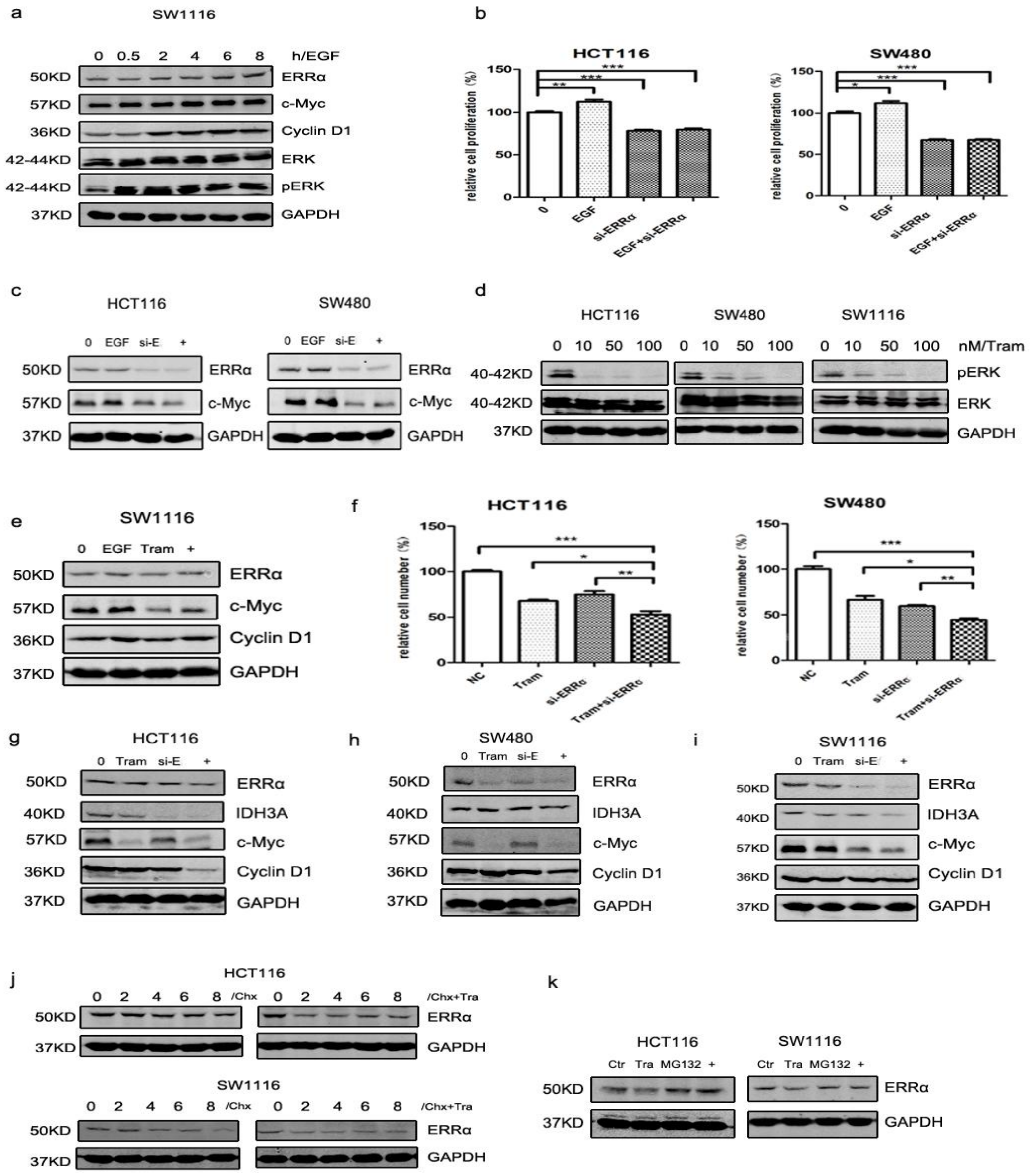

Figure S2. Suppression of ERRα completely reduces the EGF treatment-induced cell proliferation and enhances the cytotoxicity of trametinib. **a** WB for ERRα, c-Myc, cyclin D1, pERK and ERK in the SW1116 cells treated with EGF (20/μl) at the indicated times (0.5 h, 2 h, 4 h, 6 h, and 8 h) in serum-free medium. **b** CCK-8 assay for the HCT116 and SW480 cells cultured with si-NC or si-ERRα (or/and 20 ng/μl EGF) for 3 d (* P<0.05; ** P<0.01; *** P<0.001). The data are presented as the mean±SD of the experiments performed in triplicate. **c** WB for ERRα, c-Myc and cyclin D1 in the HCT116 and SW480 cells treated with si-NC or si-ERRα (or/and 20 ng/μl EGF) in serum-free medium for 48 h. **d** WB for pERK and ERK in the HCT116, SW480 and SW1116 cells treated with the indicated concentrations of trametinib (0-100 nM) or DMSO for 48 h. **e** WB for ERRα, c-Myc and cyclin D1 in the SW1116 cells treated with DMSO or 10 nM trametinib (or/and 20 ng/μl EGF) for 48 h. **f** CCK-8 assay for the HCT116 and SW480 cells treated with si-ERRα (or/and 50 nM trametinib) for 3 d**. g, h, i** WB for ERRα, IDH3A, c-Myc and Cyclin D1 in the HCT116, SW480 and SW1116 cells treated with si-ERRα (or/and 50 nM trametinib) for 2 d. **j** WB for ERRα in the HCT116 and SW1116 cells treated with cycloheximide (10 μg/ml) or trametinib combined with cycloheximide in a time-course experiment. **k** WB for ERRα in the HCT116 and SW1116 treated with trametinib (50 nM, 48 h) or DMSO supplemented with or without MG132 (10 μM) for 8 h.
