# Supplementary material for: ERRα suppression enhances the cytotoxicity of the MEK inhibitor trametinib against colon cancer cells
Source: J Exp Clin Cancer Res. 2018 Sep 5;37:218. doi: 10.1186/s13046-018-0862-8 (PMC6125878; doi:10.1186/s13046-018-0862-8)
Supplement: Supplementary file 3 — Figure S6. Quantitative RT–PCR analysis for interactions between drugs. a Quantitative real-time PCR analysis of ERRα, IDH3A and CytC in the SW480 cells treated with shNC or shERRα#2 (or/and 20 ng/μl EGF) for 2 d. b QPCR for ERRα, IDH3A and CytC in the SW480 cells treated with DMSO or trametinib (10 nM) (or/and 20 ng/μl EGF) for 2 d. c SW480 cells cultured with DMSO or 50 nM trametinib (or/and shERRα#2) at day 2 by qPCR assay. d Quantitative real-time PCR analysis of ERRα, IDH3A and CytC in the SW480 cells treated with 10 μM simvastatin (or/and 50 nM trametinib) for 2d. (PDF 949 kb) [file 13046_2018_862_MOESM3_ESM.pdf]

Additional file 3:

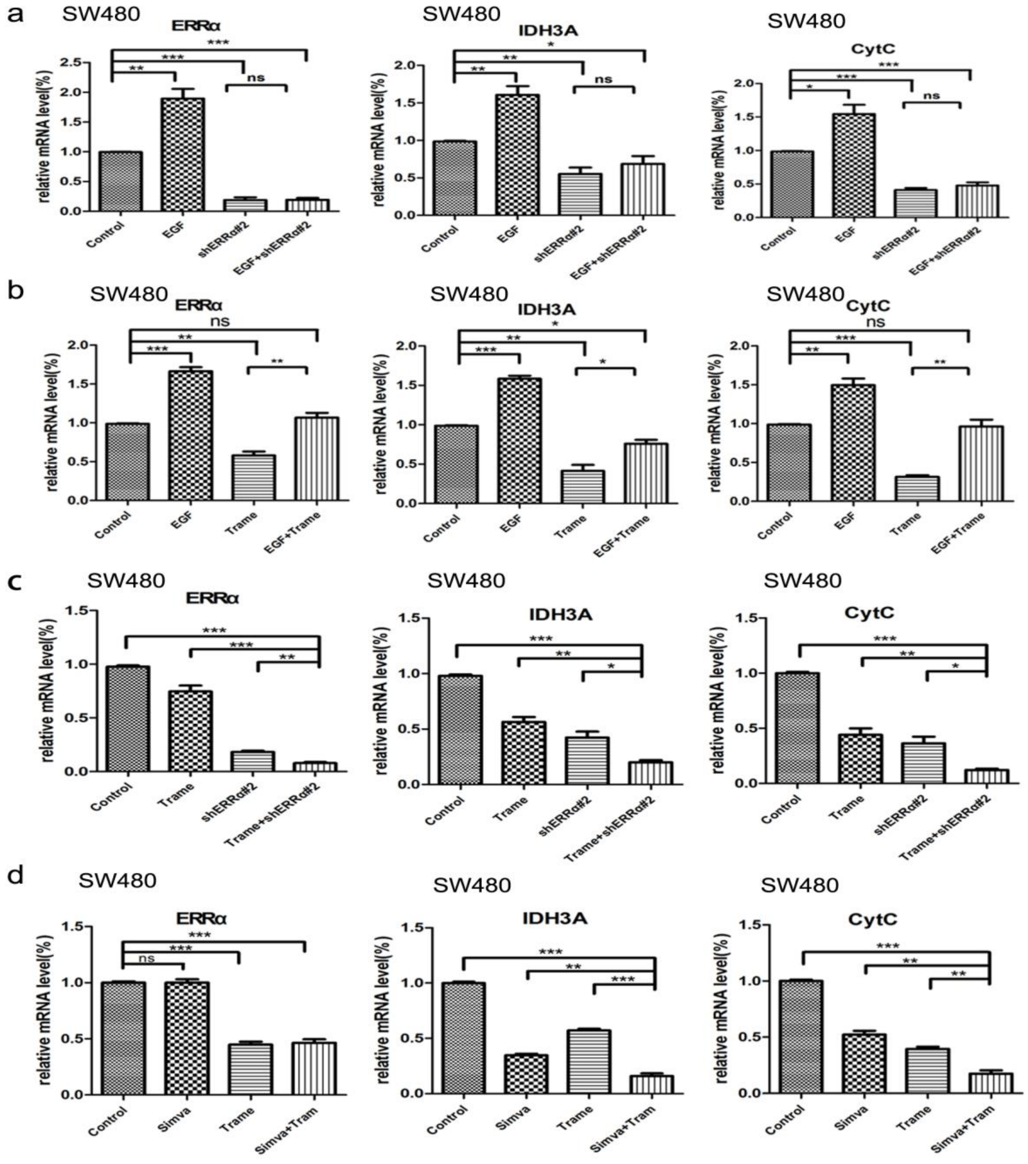

Figure S6. Quantitative RT–PCR analysis for interactions between drugs. **a** Quantitative real-time PCR analysis of ERRα, IDH3A and CytC in the SW480 cells treated with shNC or shERRα#2 (or/and 20 ng/µl EGF) for 2 d. **b** QPCR for ERRα, IDH3A and CytC in the SW480 cells treated with DMSO or trametinib (10 nM) (or/and 20 ng/µl EGF) for 2 d. **c** SW480 cells cultured with DMSO or 50 nM trametinib (or/and shERRα#2) at day 2 by qPCR assay. **d** Quantitative real-time PCR analysis of ERRα, IDH3A and CytC in the SW480 cells treated with 10 µM simvastatin (or/and 50 nM trametinib) for 2d.
