# Supplementary material for: ERRα suppression enhances the cytotoxicity of the MEK inhibitor trametinib against colon cancer cells
Source: J Exp Clin Cancer Res. 2018 Sep 5;37:218. doi: 10.1186/s13046-018-0862-8 (PMC6125878; doi:10.1186/s13046-018-0862-8)
Supplement: Supplementary file 4 — Figure S3. Simvastatin decreases the transcriptional activity of ERRα in colon cancer cells. a Cell proliferation assays at day 3 for the SW1116 cells cultured with simvastatin (5 μM and 10 μM) using the Cell Counting Kit-8. b Clonogenic assays and qualitative analysis of the SW1116 cells cultured with DMSO or 5 μM simvastatin (or/and 20 ng/μl EGF) at day 7. (PDF 947 kb) [file 13046_2018_862_MOESM4_ESM.pdf]

Additional file 4:

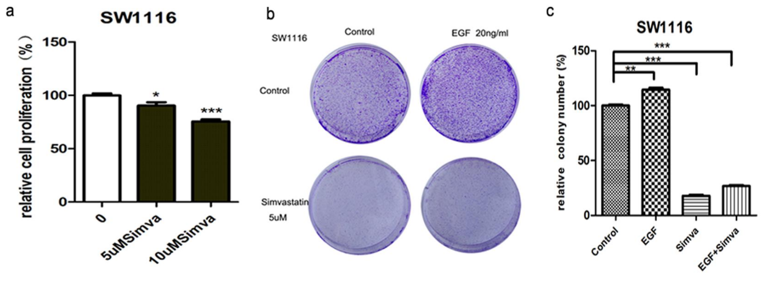

Figure S3. Simvastatin decreases the transcriptional activity of ERRα in colon cancer cells. **a** Cell proliferation assays at day 3 for the SW1116 cells cultured with simvastatin (5 μM and 10 μM) using the Cell Counting Kit-8. **b** Clonogenic assays and qualitative analysis of the SW1116 cells cultured with DMSO or 5 μM simvastatin (or/and 20 ng/μl EGF) at day 7.
