# Supplementary material for: ERRα suppression enhances the cytotoxicity of the MEK inhibitor trametinib against colon cancer cells
Source: J Exp Clin Cancer Res. 2018 Sep 5;37:218. doi: 10.1186/s13046-018-0862-8 (PMC6125878; doi:10.1186/s13046-018-0862-8)
Supplement: Supplementary file 5 — Figure S4. Antitumour effect of the combination of trametinib and simvastatin. a The CI and Fa of the HCT116 and SW480 cells treated with DMSO or 10 μM simvastatin (or/and 50 nM trametinib) for 48 h by CompuSyn software. b The tumours were analysed for proliferation (c-Myc, cyclin D1) and proapoptotic (Bax) proteins by an immunoblot assay. GAPDH was used as the protein loading control. (PDF 947 kb) [file 13046_2018_862_MOESM5_ESM.pdf]

Additional file 5:

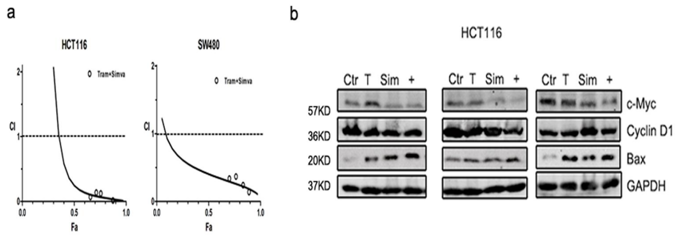

Figure S4. Antitumour effect of the combination of trametinib and simvastatin. **a** The CI and Fa of the HCT116 and SW480 cells treated with DMSO or 10 μM simvastatin (or/and 50 nM trametinib) for 48 h by CompuSyn software. **b** The tumours were analysed for proliferation (c-Myc, cyclin D1) and proapoptotic (Bax) proteins by an immunoblot assay. GAPDH was used as the protein loading control.
