# Supplementary material for: ERRα suppression enhances the cytotoxicity of the MEK inhibitor trametinib against colon cancer cells
Source: J Exp Clin Cancer Res. 2018 Sep 5;37:218. doi: 10.1186/s13046-018-0862-8 (PMC6125878; doi:10.1186/s13046-018-0862-8)
Supplement: Supplementary file 6 — Figure S5. a HMGCR is higher in carcinomatous tissues. HMGCR were identified and confirmed by Western blot analysis in 12 pairs of colon cancer tissues. (F:distal normal tissues;C:colon cancer tissues). (PDF 946 kb) [file 13046_2018_862_MOESM6_ESM.pdf]

Additional file 6:

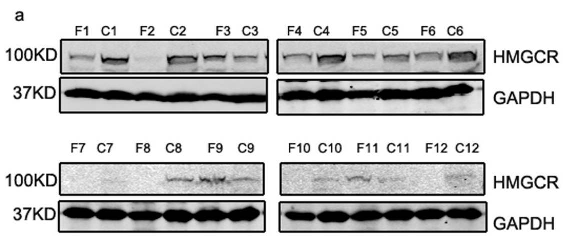

Figure S5. a HMGCR is higher in carcinomatous tissues. HMGCR were identified and confirmed by Western blot analysis in 12 pairs of colon cancer tissues. (F:distal normal tissues;C:colon cancer tissues)
